# Urokinase-type plasminogen activator receptor interaction with β1 integrin is required for platelet-derived growth factor-AB-induced human mesenchymal stem/stromal cell migration

**DOI:** 10.1186/s13287-015-0163-5

**Published:** 2015-09-29

**Authors:** Valérie Chabot, Cécile Dromard, Angélique Rico, Alain Langonné, Julien Gaillard, Fabien Guilloton, Louis Casteilla, Luc Sensebé

**Affiliations:** EFS Centre-Atlantique, BP 40661, 37 206 Tours, Cedex 3 France; CNRS UMR5273 STROMALab, BP 84225, F-31 432 Toulouse, Cedex 4 France; Université Paul Sabatier de Toulouse, BP 84225, F-31 432 Toulouse, Cedex 4 France; INSERM U1031, BP 84225, F-31 432 Toulouse, Cedex 4 France; EFS Pyrénées –Méditerranée BP 84225, F-31 432 Toulouse, Cedex 4 France; Département des Microscopies, Faculté de Médecine, 37 032 Tours, Cedex France

## Abstract

**Introduction:**

Mesenchymal stem cells (MSC) are well described for their role in tissue regeneration following injury. Migratory properties of endogenous or administrated MSC are critical for tissue repair processes. Platelet-derived growth factor (PDGF) is a chemotactic growth factor that elicits mesenchymal cell migration. However, it is yet to be elucidated if signaling pathways other than direct activation of PDGF receptor (PDGF-R) are involved in PDGF-induced cell migration.

**Methods:**

Knocking down and co-immunoprecipitation approaches were used to evaluate urokinase-type plasminogen activator receptor (uPAR) requirement and its interactions with proteins involved in migration mechanisms, in human MSC induced to migrate under PDGF-AB effect.

**Results:**

We demonstrated that uPAR activation and its association with β1-integrin are required for PDGF-AB-induced migration. This phenomenon takes place in MSC derived from bone marrow and from adipose tissue.

**Conclusions:**

We showed that PDGF-AB downstream signaling requires other effector molecules in MSC such as the uPA/uPAR system and β1 integrin signaling pathway known for their role in migration. These findings provide new insights in molecular mechanisms of PDGF-AB-induced migration of human MSC that may be relevant to control MSC function and tissue remodeling after injury.

**Electronic supplementary material:**

The online version of this article (doi:10.1186/s13287-015-0163-5) contains supplementary material, which is available to authorized users.

## Introduction

Mesenchymal stem cells (MSC) were first identified in bone marrow as a population of plastic adherent and highly proliferative cells that were able to form colonies of fibroblasts (colony-forming unit-fibroblasts) [1] and display multipotency towards multiple lineages *in vitro *and *in vivo *upon transplantation (cartilage, bone, fat, vascular tissue, and muscle) [2, 3]. Moreover, bone marrow mesenchymal stem cells (BM-MSC) show potent trophic and immunomodulatory functions that promote tissue repair [4–7]. Nevertheless, the ability of locally injected or mobilized MSC to migrate *in vivo *to the sites of injury remains a critical step. Several studies have demonstrated the homing of administered MSC to sites of injury [8, 9]; the migration of MSC to such sites may be dependent on chemotactic signals derived from injured or inflamed tissues. A large range of factors exert chemotactic effects on MSC including inflammatory cytokines such as interleukin (IL)-1β and tumor necrosis factor alpha (TNFα) and growth factors [10–12] that can have additive effects [12]. The platelet-derived growth factors (PDGFs) are released following platelet aggregation early after tissue injury and during the inflammatory process [13]. Numerous studies have demonstrated an important role for these factors in migration, extracellular matrix (ECM) synthesis, and proliferation of mesenchymal cells, making them key regulatory molecules of tissue repair [14], but Ponte et al. [12] showed that TNFα does not enhance MSC migration towards PDGF. In both mouse and human, the PDGFs consist of four inactive monomeric ligands (A–D) that assemble into biologically active homodimers; only PDGF-A and PDGF-B can form functional heterodimers. Although PDGF-AA and PDGF-BB cell signaling has been well described on migration of cells of mesenchymal origin [14, 15], less is known about the effects of PDGF-AB. The PDGF-AB heterodimer has been identified in platelets and may be specific to humans. Cell stimulation with PDGF-AB leads to preferential formation of heterodimer receptors (PDGFR-αβ) and increases its mitogenicity compared with PDGF-AA or PDGF-BB. The PDGF-AB heterodimer is thus endowed with different signaling properties from the homodimers (for review see [16]). PDGF-AB was also shown to promote human MSC migration [12, 17], but many questions related to the molecular mechanisms underlying its effects remain largely unanswered.

The receptor of the urokinase-type plasminogen activator (uPA) serine protease is present on the surface of many cell types, including myeloid cells, endothelial cells, epithelial cells, and vascular smooth muscle cells (VSMC). Initially described as regulating hemostasis [18], urokinase-type plasminogen activator receptor (uPAR) has since been shown to be overexpressed upon cell exposure to inflammatory mediators and to play a critical role in the regulation of various cell properties, especially cell migration (for review see [19]). Indeed, numerous studies reported that the receptor strongly contributes to tumor invasiveness (for review see [20]) and VSMC migration [21–25], and Carlin et al. [26] found that uPA and PDGF have an additive effect on airway smooth muscle cell migration. Recently, it was shown that uPA/uPAR also plays a pivotal role in regulating migration and differentiation of MSC [27–31]. Vallabhaneni et al. [29] demonstrated the involvement of uPAR in mobilization of MSC from mouse bone marrow as well as uPAR requirement for engraftment of injected human BM-MSC to the vessel wall of injured mouse femoral arteries. uPAR modulates migration through cooperation between extracellular proteolysis and intracellular signaling. The human uPAR protein displays three extracellular domains. The amino-terminal domain (D1) contains the main binding site for uPA. Once bound, uPA catalyzes the conversion of plasminogen into plasmin that activates several matrix metalloproteinases, thus promoting localized ECM degradation including fibronectin and vitronectin. Independently of its extracellular proteolytic role, uPA/uPAR regulates cell migration through activation of intracellular signaling pathways. uPAR is associated with the plasma membrane by a glycosyl-phosphatidylinositol anchor but lacks cytoplasmic domains. Consequently, it requires transmembrane coreceptors for intracellular signaling. The ECM protein receptors integrins are the most important transmembrane partners physically and functionally associated with uPAR able to connect uPAR to intracellular signaling pathways [32]. Interestingly, it has recently been shown that integrins are highly expressed on MSC and that β1 integrin is the most abundant β integrin on the surface of MSC [33, 34].

In this work, we provide evidence that PDGF-AB triggers human BM-MSC migration by recruiting uPAR and β1 integrins, thus promoting their colocalization at the cell surface and driving promigratory signaling pathways. Moreover, we show that this regulatory system is not restricted to MSC purified from human bone marrow but also triggers migration of an additional MSC cell type; that is, adipose tissue-derived MSC [35].

## Methods

### *In vitro* production and characterization of MSC

Human BM-MSC were isolated from bone marrow of seven healthy donors. Bone marrow was aspirated from the posterior iliac crest of adults undergoing orthopedic surgery (Orthopedic Surgery Department, Trousseau Hospital, Tours, France) after approval from the Medical Ethics Committee of Tours (“Comité de Protection des Personnes Tours—CPP Région Centre [Ouest-1]”) and in accordance with their guidelines. Written informed consent was obtained from all patients for the use of their samples. Adipose stem/stromal cells (ASC) were isolated as described previously [36] from subcutaneous adipose tissue obtained from nonobese patients undergoing elective abdominal dermolipectomy (Plastic Surgery Department, Rangueil Hospital, Toulouse, France). No objection certificates were obtained, according to Bioethics Law No. 2004–800 of 6 August 2004. These tissue samples are seen as waste biological samples in France and do not require informed consent for their use, in accordance with the French ethical and legal regulations.

Adherent cells were grown *in vitro *in minimum essential medium alpha (MEMα; Invitrogen, Cergy-Pontoise, France) supplemented with 10 % fetal calf serum (FCS; Perbio, Brébières, France), 2 mM l-glutamine (Invitrogen), 100 U/ml penicillin (Invitrogen), 10 μg/ml streptomycin (Invitrogen), 2.5 μg/ml fungizone (Bristol-Myers-Squibb, Rueil-Malmaison, France) and 1 ng/ml fibroblast growth factor-2 (FGF-2; R&D Systems, Lille, France) as described previously [37]. Following passage 1, when cultured cells achieved 80–90 % confluence, they were trypsinized as follows: culture medium was removed, cells were washed twice with 1X phosphate-buffered saline (PBS; Invitrogen) and a solution containing 0.05 % trypsin and 0.53 mM ethylenediamine tetraacetic acid (EDTA; Invitrogen) was added. The cells were incubated at room temperature for 5 minutes, and afterwards trypsin was neutralized by the addition of serum. Cells were counted and viability was assessed using Trypan blue. Flow cytometry analysis indicated that these cells expressed CD105, CD73, and CD90 and lacked expression of CD45, CD34, CD11b, CD19, and HLA-DR (Figure S1A in Additional file 1). As described previously, these cells displayed multipotent differentiation potential under standard *in vitro *differentiation conditions (Figure S1B in Additional file 1). Detailed protocols for cell differentiation are presented in Additional file 2.

### BM-MSC stimulation by inflammatory cytokines and PDGF-AB

Passage 1 adherent cells were grown for 5–24 hours (depending on experiments), at 37 °C in serum-free control medium (RPMI (Invitrogen), 0.25 % bovine serum albumin (BSA; PAA Laboratories, Velizy-Villacoublay, France)) or treated with IL-1β (0.2 ng/ml), IL-8 (150 ng/ml), TNFα (10 ng/ml), or PDGF-AB (50 ng/ml) (R&D Systems). Cells were dissociated using trypsin–EDTA solution (Invitrogen) for PCR analysis or Cell Dissociation Solution (Sigma-Aldrich, St Quentin Fallavier, France) for flow cytometry and western blot analysis.

### Migration assays

#### Scratch test

Passage 1 BM-MSC and ASC were serum starved by culturing them in serum-free control medium for 24 hours. Cells were detached and 1 × 10^5^ cells were seeded in 12-well plates (Corning, Cambridge, MA, USA) coated or not with 10 μg/ml type I dermal collagen from human skin (Merck Millipore-Calbiochem, Saint Quentin en Yvelines, France) or 5 μg/ml human cellular fibronectin (Sigma-Aldrich, Saint Quentin-Fallavier, France) or 500 ng/ml human vitronectin (BD Biosciences, Le Pont de Claix, France ). After 2 hours of adherence at 37 °C, the scratches were performed using P200 pipette tips and pictures were immediately taken (T = 0) using an inverted microscope (TG2000-S; Nikon, Champigny sur Marne, France) and a camera (DXM1200F; Nikon).

The cells were then treated or not with IL-1β (0.2 ng/ml), IL-8 (150 ng/ml), TNFα (10 ng/ml), or PDGF-AB (50 ng/ml) at 37 °C. Pictures of scratches were taken again after 14, 18, and/or 22 hours to visualize the wound closure. Scratched surfaces were measured using ImageJ software [38] (National Institute of Health, Bethesda, MD, USA). Wound closure surfaces were calculated as the scratch surface at T = 0 minus the scratch surface at T = 22 hours, and results were expressed as percentages of wound closure (wound closure surface/scratch surface at T = 0)x 100.

For inhibitory experiments, before adding neutralizing antibodies, surface uPA was acid-stripped from adherent BM-MSC by incubation of cells with 0.05 M glycine, 0.1 M NaCl (pH 3.0; 2 minutes at room temperature), neutralized with 0.5 M HEPES, 0.1 M NaCl (pH 7.5), and washed with RPMI. Cells were then pretreated or not with 5 μg/ml goat anti-human uPAR (R&D Systems), 1.5 μg/ml mouse anti-human uPA (American Diagnostica, Neuville sur Oise, France) neutralizing antibodies, or 50 μM integrin blocking peptide β1P1 (NLDSPEGGF; Tebu-Bio, Le Perray en Yvelines, France) for 30 minutes at 37 °C along with corresponding controls: IgG_1_ and the scrambled peptide sc β1P1 (EDGLFNPSG; Tebu-Bio). Afterwards, cells were treated or not with 50 ng/ml PDGF-AB. Pictures of scratches were taken again to visualize wound closure.

#### Transwell migration

Detailed protocols are presented in Additional file 2.

### Proliferation assay

Detailed protocols are presented in Additional file 2.

### Quantitative (real-time) PCR

We quantified uPAR and uPA mRNA in BM-MSC and ASC treated or not with IL-8, TNFα, or PDGF-AB for 5, 10, or 24 hours. BM-MSC treated with 100 ng/ml phorbol myristate acetate (PMA; Sigma-Aldrich) for 18 hours at 37 °C were used as a positive control for uPAR and uPA mRNA expression. Total RNA was isolated using the RNeasy Kit (Qiagen, Courtaboeuf, France), according to the manufacturer’s instructions. First-strand cDNA was synthesized from 1.5–2.0 μg total RNA using the PrimeScript™ first strand cDNA synthesis Kit (TaKaRa, Saint Germain en Laye, France). All samples were subjected to PCR amplification with oligonucleotide primers for uPAR or uPA and the constitutively expressed gene for GAPDH. PCR products were fluorescently labeled using the Eva Green SMX 1000 (Biorad, Marnes La Coquette, France) and real-time monitoring of PCR products was measured by fluorescence (SYBR green) with the Light Cycler (Biorad). Normalization to GAPDH expression and quantification of RT-PCR were performed using Light Cycler software (Biorad). A significant change in gene expression after PDGF-AB treatment was considered when results were concordant in three experiments showing at least a twofold increase or decrease compared with baseline values.

The primer sequences used for real-time PCR are listed in Additional file 2.

### Flow cytometry

Detailed protocols are presented in Additional file 2.

### Preparation of BM-MSC cellular membranes

BM-MSC grown in serum-free control medium (RPMI–0.25 % BSA) or treated with PDGF-AB (50 ng/ml) were detached and centrifuged for 10 minutes at 400 × *g*. After washing with ice-cold PBS, the number and the viability of the cells were estimated with Trypan blue. Cell pellets were used immediately for preparation of cellular membranes as described previously [39], with minor modifications. Briefly, the pellets were resuspended in ice-cold Dounce buffer (10 mM Tris–HCl, pH 7.6, 0.5 mM MgCl_2_, 1 mM phenylmethylsulfonyl fluoride (PMSF; Sigma-Aldrich), protease inhibitor cocktail (Sigma-Aldrich)) and homogenized using Dounce homogenizer. Cell suspensions were centrifuged at 4 °C for 5 minutes at 400 × *g* to remove the nuclear fraction. The supernatants were collected and centrifuged at 100,000 × *g* at 4 °C for 1 hour to isolate the membranes. BM-MSC cellular membranes were kept at −80 °C until analysis by western blot and zymography. Total protein content was measured using the Coo protein assay determination kit (Uptima, Montluçon, France).

### Western blot analysis of BM-MSC cellular membranes

Detailed protocols are presented in Additional file 2.

### Zymography of BM-MSC cellular membranes

Detailed protocols are presented in Additional file 2.

### Small interfering RNA and cell transfection

Passage 0 BM-MSC and ASC were transfected with small interfering RNA (siRNA) of either nontargeting control siRNA (si Neg) or uPAR siRNA (siPLAUR5-6; Qiagen) using the Amaxa™ Cell Line Nucleofector™ kit V (Lonza, Levallois-Perret, France) according to the manufacturer’s recommendations. Then 500,000 cells were suspended in 100 μl Nucleofector^®^ solution (Lonza) and mixed with 1.5 μg siRNA. Samples were transferred into the Nucleofector^®^ machine and transfected using the T030 transfection program. Cells were transferred to culture dishes and grown in expansion medium (MEMα, 10 % FCS, 2 mM l-glutamine, 100 U/ml penicillin, 10 μg/ml streptomycin, 2.5 μg/ml fungizone, and 1 ng/ml FGF-2) until they reached 80 % confluence 5 days later.

### Confocal fluorescence microscopy

The cellular localization of uPAR, actin-F, phosphorylated focal adhesion tyrosine kinase (P-FAK Tyr^397^), and β1-integrin subunit on migrating BM-MSC were analyzed by confocal microscopy. After a 24-hour serum starvation, cells were detached and seeded in Lab-Tek^®^ two-chamber slides (Nalge Nunc International, Rochester, NY, USA) coated with 10 μg/ml type I dermal collagen or 5 μg/ml cellular fibronectin. After 2 hours of adherence, scratches were performed and cells were grown in serum-free control medium or treated with 50 ng/ml PDGF-AB for 1, 3, or 6 hours.

Immunocytochemistry detection was performed after fixation with 4 % paraformaldehyde in PBS for 15 minutes at room temperature. Nonspecific binding sites were blocked with 5 % normal goat serum and cells were incubated with an anti-uPAR antibody (10 μg/ml mouse anti-human uPAR 3936 or polyclonal rabbit anti-human uPAR 399R; both American Diagnostica) for 1 hour at room temperature followed respectively by incubation with Alexa-488-conjugated goat anti-mouse or anti-rabbit antibody (1:1000; Molecular Probes-Invitrogen, Cergy Pontoise, France) for 30 minutes. Cells were permeabilized with 0.2 % Tween 20 and incubated respectively with AlexaFluor 594-phalloïdin (1:40; Molecular Probes-Invitrogen) for 30 minutes or anti P-FAK Tyr^397^ rabbit polyclonal antibody (1:50; Santa Cruz Biotechnologies, Heidelberg, Germany) for 1 hour followed by Alexa-594-conjugated goat anti-rabbit antibody (1:1000; Molecular Probes-Invitrogen) for 30 minutes. For β1-integrin staining, cells were incubated with anti-human integrin β1 (JB1A, 1:400; Chemicon-Millipore, Guyancourt, France) mouse antibody for 1 hour followed by Alexa-594-conjugated goat anti-mouse antibody (1:1000; Molecular Probes-Invitrogen) for 30 minutes.

Samples were imaged after mounting with Dako mounting medium which stains nuclei with 4′,6-diamidino-2-phenylindole (DAPI). Confocal microscopy imaging was performed on an inverted laser-scanning Olympus FV500 microscope (Olympus, Rungis, France ). Images were captured with a ×60 oil-immersion objective, analyzed with Olympus software, and were subsequently processed for analysis using Imaris software (Bitplane AG, Zurich, Switzerland).

### Immunoprecipitation and western blot analysis

To determine uPAR/P-FAK and uPAR/β1 integrin associations, coimmunoprecipitation experiments were performed. Serum-starved cells were seeded in six-well plates coated with 10 μg/ml type I dermal collagen or 5 μg/ml cellular fibronectin. After 2 hours of adherence, cells were treated or not with 50 ng/ml PDGF-AB for 1, 3, or 18 hours and lysed on ice with RIPA buffer (Santa Cruz Biotechnologies) supplemented with protease inhibitor cocktail, 2 mM PMSF (Sigma-Aldrich), and phosphatase inhibitor cocktails (Santa Cruz Biotechnologies), at each time point; that is, before adherence (T0), 2 hours post adherence (Adh), and following PDGF-AB treatment (during 1, 3, and 18 hours). The insoluble fraction was pelleted by centrifugation at 13,000 rpm for 10 minutes at 4 °C and total protein content was measured using the Coo protein assay determination kit (Uptima).

The supernatants (100 μg proteins) were precleared with protein A/G Plus agarose beads (Santa Cruz Biotechnologies) for 2 hours at 4 °C, and then incubated overnight at 4 °C with antibodies against integrin β1 (JB1A, 1:1000; Chemicon-Millipore) for β1 integrin/uPAR coimmunoprecipitation experiments, or uPAR (IID7, 2 μg; Santa Cruz Biotechnologies) for uPAR/P-FAK coimmunoprecipitation experiments, or isotypic control IgG. Immunoprecipitates were washed with PBS, eluted with Laemmli sample buffer, resolved by 8 % (for β1 integrin) or 12 % (for uPAR) SDS-PAGE and then blotted for β1 integrin (1:200; Chemicon-Millipore) and uPAR (399R, 1:1000; American Diagnostica) or uPAR (399R, 1:1000; American Diagnostica) and P-FAK (1:1000; Invitrogen). After being washed, PVDF membranes were incubated with horseradish peroxidase (HRP)-conjugated goat anti-mouse IgG or HRP-conjugated goat anti-rabbit IgG (1:3000; Biorad). Protein bands were revealed by chemiluminescence using the ECLplus western blotting reagent (GE Healthcare Europe GmbH, Aulnay sous Bois, France) and membranes were analyzed at 430 nm using a camera and the Chemicapt^®^ software (Vilber Lourmat , Marne la Vallée, France). Bands were quantified by densitometry analysis using ImageJ software (National Institute of Health).

### Statistical analysis

Data are expressed as mean ± standard error of the mean (SEM). They were analyzed with GraphPad Prism 5 software (La Jolla, CA, USA ) using the parametric *t* test or the nonparametric Mann–Whitney test with the significance level set at *P* <0.05.

## Results

### PDGF-AB enhances BM-MSC migration on type I dermal collagen, cellular fibronectin, and vitronectin

**Fig. 1:**
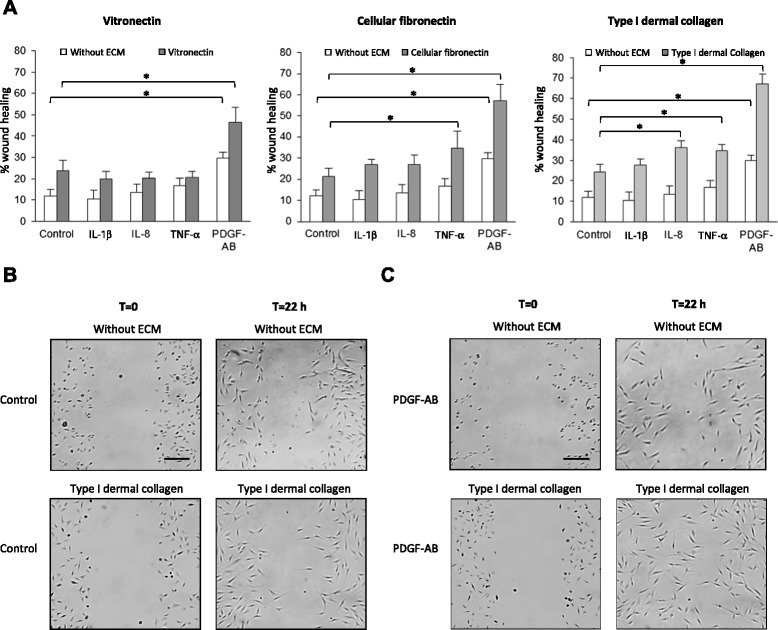
Regulation of BM-MSC migration by inflammatory molecules. Scratch test assay was performed on BM-MSC isolated from three different donors and seeded in plates coated with type I dermal collagen, cellular fibronectin, or vitronectin or without ECM. Pictures of scratches were taken immediately (T = 0) or 22 hours after culture (T = 22 h) in serum-free control medium or medium supplemented with IL-1β, IL-8, TNFα, or PDGF-AB. **a** Quantification of BM-MSC migration. Results expressed as percentages of wound closure at T = 22 h compared with T = 0 for each condition. Data expressed as mean ± SEM of three independent experiments (one donor per experiment), each performed in triplicate. *P <0.05. **b, c** One experiment representative of the three experiments is shown. *ECM* extracellular matrix, *IL* interleukin, *PDGF-AB* platelet-derived growth factor AB, *TNFα* tumor necrosis factor alpha

To investigate which inflammatory molecules play a key role in BM-MSC migration, we used an *in vitro *scratch test assay in which plates were coated or not with three different ECM proteins overexpressed in wounding tissues including vitronectin, fibronectin, and type I dermal collagen. In the presence or absence of ECM, nontreated BM-MSC displayed poor migratory capacities 22 hours after the scratch (Control; Fig. 1a, b). IL-1β, IL-8, and TNFα had no effect on BM-MSC migration in the absence of ECM or on vitronectin, compared with nontreated BM-MSC (Control; Fig. 1a). IL-1β did not affect BM-MSC migration in any other conditions tested while IL-8 stimulated BM-MSC migration on collagen I only (12 % enhancement) and TNFα increased BM-MSC migration on fibronectin (13 % enhancement) and collagen I (10 % enhancement). The effects of IL-8 and TNFα on BM-MSC migration were far less than those of PDGF-AB. PDGF-AB significantly enhanced the migration of BM-MSC; 18 % enhancement in absence of ECM, 22 % on vitronectin, 36 % on fibronectin, and greater effects were observed on type I dermal collagen: 43 % enhancement (Fig. 1a, c). As migratory stimulation by PDGF-AB was observed for the three time points we tested (14, 18, and 22 hours) (data not shown), all of the following migration experiments were conducted 22 hours after scratch. We controlled so that proliferation of BM-MSC was not increased under any condition used for the scratch test assay (Figure S2A in Additional file 3). The effect of PDGF treatment on stimulation of MSC migration was confirmed using another migration assay, in transwell dishes (Figure S2B in Additional file 3). The results showed that PDGF-AB treatment for 22 hours increased by sixfold the number of cells that migrated on the lower face of the filters. These results indicate that BM-MSC migrate in response to several molecules known to be released in damaged tissue and that PDGF-AB is one of the most potent, especially on collagen I.

### PDGF-AB stimulates uPAR and uPA expression in BM-MSC

**Fig. 2:**
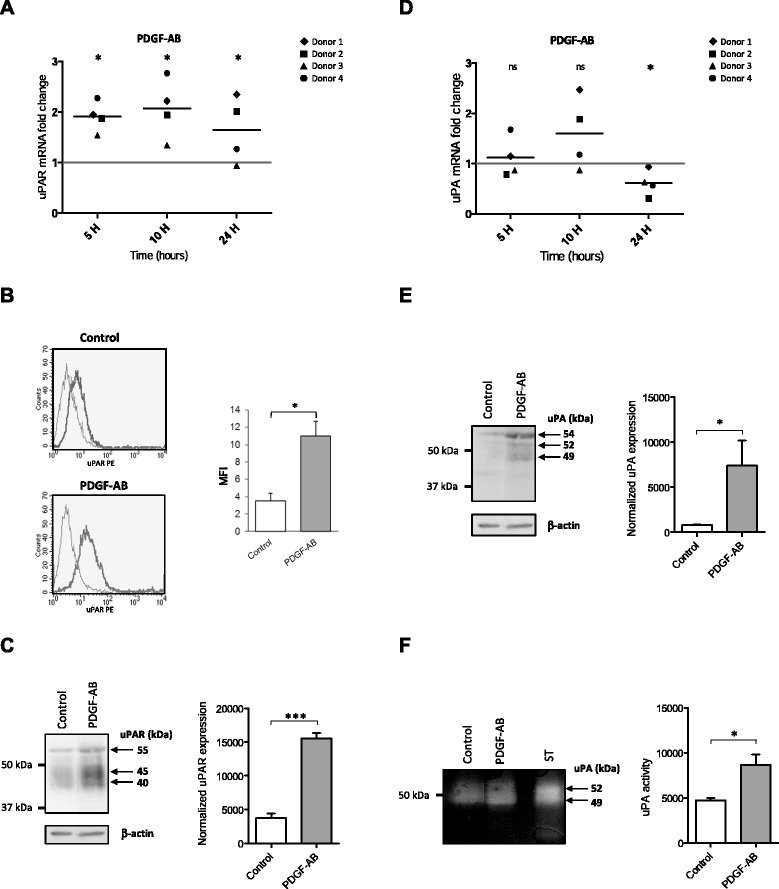
PDGF-AB regulates uPAR and uPA expression in BM-MSC. BM-MSC were grown in serum-free control medium or treated with PDGF-AB. **a** Quantitative PCR analysis of uPAR mRNA synthesis 5, 10, or 24 hours after treatment. Data from four donors are presented as the uPAR mRNA fold change relative to nonstimulated control cells for each donor (1 = no stimulation). **b** Flow cytometry analysis of uPAR cell surface expression; 24 hours after treatment, cells were stained with anti-uPAR monoclonal antibody (*dark curves*) or its isotypic control (*grey curves*). One representative experiment of one donor of the five assessed is shown (*left*). Quantification data (*right*) expressed as mean ± SEM of uPAR fluorescence intensity relative to the isotype of five independent experiments (one donor per experiment). *P <0.05. **c** Western blot analysis of uPAR in cellular membranes 24 hours after treatment.***P <0.001 **d** Quantitative PCR analysis of uPA mRNA synthesis 5, 10, or 24 hours after treatment. Data from four donors are presented as uPA mRNA fold change relative to nonstimulated control cells for each donor (1 = no stimulation). **e** Western blot analysis of uPA in cellular membranes 24 hours after treatment. **f** Zymographic analysis of uPA activity in cellular membranes 24 hours after treatment. Purified high molecular weight human urokinase was used as standard (ST). **c**, **e**, **f** (*left*) Data represent one representative experiment, (*right*) bands were quantified by densitometry analysis using ImageJ software, normalized to β-actin (**c**, **e**). Data expressed as mean ± SEM of three to five independent experiments (one donor per experiment). *MFI* mean fluorescence intensity, *PDGF-AB* platelet-derived growth factor AB, *PE* phycoerythrin, *uPA* urokinase-type plasminogen activator, *uPAR* urokinase-type plasminogen activator receptor

To examine whether uPAR was involved in PDGF-AB-induced migratory capacities, we first quantified uPAR gene expression in BM-MSC and its regulation by PDGF-AB. Kinetic experiments revealed that uPAR mRNA levels reached a twofold increase after treatment with PDGF-AB as soon as 5 hours post treatment (Fig. 2a). At the protein level, both flow cytometry analysis on intact cells and western blotting on isolated cell membrane fractions revealed that control BM-MSC weakly expressed uPAR at their surface, whereas 24 hours post PDGF-AB treatment the cell surface expression of uPAR was significantly increased (Fig. 2b, c), indicating that mRNA and protein expressions are regulated in a similar manner. Three major isoforms of uPAR were detected in BM-MSC cellular membranes at 55, 45, and 40 kDa and each isoform’s expression was increased by the PDGF-AB treatment (Fig. 2c).

Because uPAR could be activated in an autocrine manner, we examined the effect of PDGF-AB on uPA mRNA gene expression in BM-MSC. In basal conditions, control MSC expressed high mRNA levels of uPA (data not shown). Following PDGF-AB treatment, uPA mRNA content tended to be upregulated at 10 hours post treatment and decreased after 24 hours (Fig. 2d). As shown in Fig. 2e, uPA was weakly detected by western blotting on cell membrane fraction of control BM-MSC, while three major bands of membrane-bound uPA (54, 52, and 49 kDa) were increased after 24 hours of PDGF-AB treatment. Zymographic analysis of the BM-MSC cell membrane fraction (Fig. 2f) confirmed that the 52 and 49 kDa bands were the active forms of uPA while there was no band detected at 54 kDa. These results highlight an activation of the uPA/uPAR system after PDGF-AB treatment. As IL-8 and TNFα induced BM-MSC migration (although to a lesser extent than PDGF-AB), uPA and uPAR gene expression were also assessed under IL-8 and TNFα treatment (Additional file 4). IL-8 did not affect uPAR or uPA gene expression. While TNFα enhanced uPAR gene expression after 24 hours of treatment (twofold increase), it strongly decreased uPA gene expression (more than 70 % reduction) compared with nontreated control conditions. These results suggest that IL-8 and TNFα stimulate BM-MSC migration via a signaling pathway that does not involve uPA.

### The uPA/uPAR system is required for PDGF-AB-induced BM-MSC migration

**Fig. 3:**
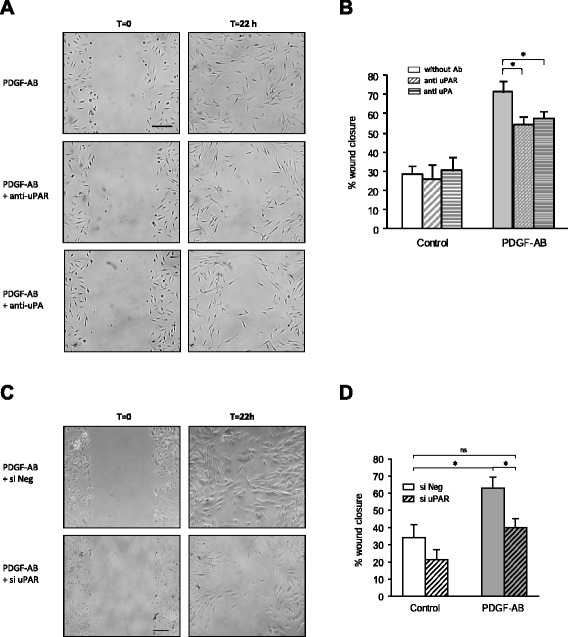
uPA/uPAR mediate PDGF-AB-induced BM-MSC migration. **a, b** Scratch test assay was performed on BM-MSC isolated from three different donors and seeded in plates coated with type I dermal collagen. Pictures of scratches were taken immediately (T = 0) or 22 hours after culture (T = 22 h) in serum-free control medium or PDGF-AB supplemented medium in the presence or not of anti-uPAR or anti-uPA neutralizing antibodies. **a** An experiment representative of three experiments. **b** Quantification of BM-MSC migration. **c, d** Scratch test assay was performed on BM-MSC isolated from three different donors transfected with either control siRNA (si Neg) or uPAR siRNA (si uPAR) and grown on type I dermal collagen in serum-free control medium or treated with PDGF-AB. **c** An experiment representative of three experiments. **d** Migration quantification of transfected BM-MSC. **b, d** Results expressed as percentages of wound closure at T = 22 h compared with T = 0. Mean of three independent experiments ± SEM are represented (one donor per experiment), each performed in triplicate. *P <0.05. Scale bars: 200 μm. *PDGF-AB* platelet-derived growth factor AB, *si Neg* nontargeting small interfering RNA, *si uPAR* uPAR-targeted small interfering RNA, *uPA* urokinase-type plasminogen activator, *uPAR* urokinase-type plasminogen activator receptor

As PDGF-AB stimulated both uPA and uPAR expression and BM-MSC migration *in vitro*, we tested whether these proteins were required for PDGF-AB-induced BM-MSC migration using two loss-of-function strategies. We first investigated the effect of neutralizing anti-uPAR antibodies in a scratch test assay. Neutralizing antibodies displayed no effect on BM-MSC basal migration but significantly decreased the capacity of PDGF-AB-treated BM-MSC to reconstitute the scratched area (Fig. 3a, b). The same results were obtained using anti-uPA antibodies (Fig. 3a, b) independently of the matrix tested (Additional file 5). To confirm these results, we used a siRNA to reduce uPAR expression in BM-MSC and the scratch test assay was performed on type I dermal collagen. BM-MSC were transfected with control siRNA (si Neg) or uPAR targeted siRNA (si uPAR) and the knocking down efficiency was evaluated by quantitative PCR and flow cytometry approaches. A 77 % decrease in uPAR mRNA expression and a 90 % decrease in uPAR protein synthesis were observed (Figure S5A, B in Additional file 6). BM-MSC wound closure capacity was increased by 29 % after 22 hours of treatment with PDGF-AB and this effect was inhibited when BM-MSC were transfected with uPAR siRNA (Fig. 3c, d). On transwell dishes, the effect of PDGF-AB was significantly reduced by uPAR siRNA (81 % reduction) (Figure S5C in Additional file 6). All together these results demonstrate that uPAR is required for PDGF-AB-induced migration of BM-MSC.

### PDGF-AB enhances migration of BM-MSC through control of uPAR and β1-integrin association

**Fig. 4:**
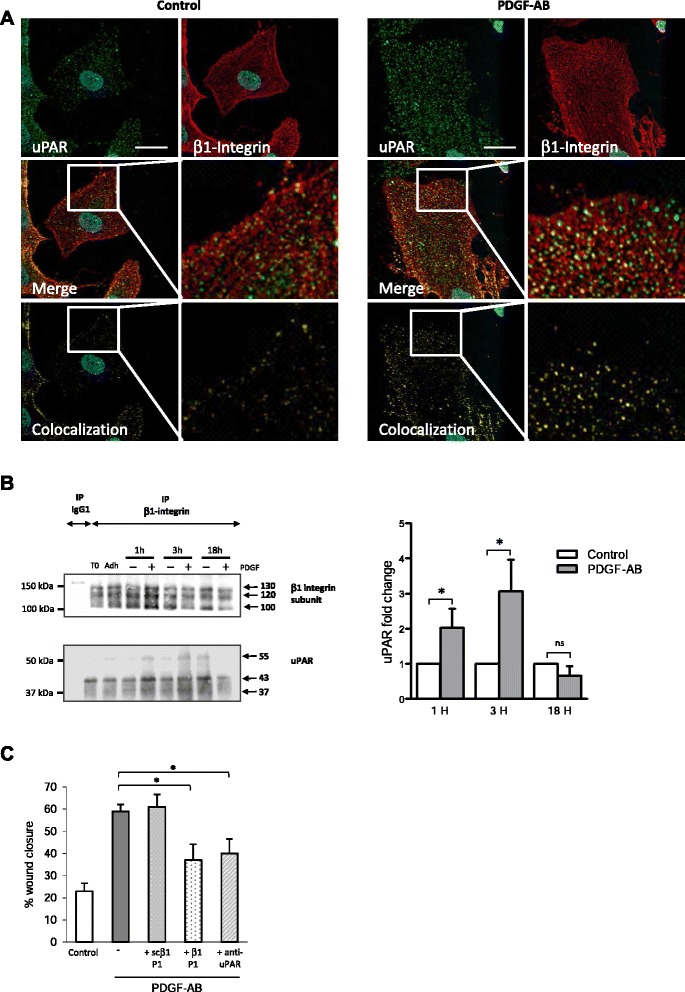
PDGF-AB enhances migration of BM-MSC through control of uPAR and β1-integrin association. **a** uPAR (*green*) and β1-integrin subunit (*red*) repartition in migrating cells. Images were acquired with a confocal microscope. uPAR and β1-integrin stainings were merged to show colocalization (*yellow*). Nuclei were stained with DAPI. Scale bars: 10 μm. **b** uPAR-β1 subunit coimmunoprecipitation. Two hours after adherence (Adh) on type I dermal collagen, cells were grown in serum-free control medium (−) or treated with PDGF-AB (+) for 1–18 hours. β1 integrin was immunoprecipitated from cell lysates with anti-β1 monoclonal antibodies or isotypic control IgG_1_. Immunoprecipitates were analyzed by SDS-PAGE and blotted for β1-integrin subunit (*top western blot*) and uPAR (*bottom western blot*). (*Left*) Western blot representative of three experiments. T0, lysate of cells before seeding; Adh., lysate of cells allowed to adhere for 2 hours on type I dermal collagen. (*Right*) uPAR blots quantification by densitometry analysis using ImageJ software. Data expressed as the uPAR fold change after PDGF-AB treatment compared with serum-free control medium from three independent experiments (one donor per experiment). **c** Scratch test assay was performed on BM-MSC isolated from three different donors and seeded in plates coated with type I dermal collagen. Two hours after adherence, cells were grown in serum-free control medium or treated with PDGF-AB, supplemented or not with integrin blocking peptide β1P1 along with the corresponding scrambled control scβ1P1 or anti-uPAR neutralizing antibody. Results are expressed as percentages of wound closure at T = 22 h compared with T = 0. Mean of three independent experiments ± SEM are represented (one donor per experiment), each performed in triplicate. *P <0.05. *PDGF-AB* platelet-derived growth factor AB, *uPAR* urokinase-type plasminogen activator receptor

We tested whether PDGF-AB promotes the association of uPAR with β1 integrin in BM-MSC. We first examined the localization of uPAR using confocal microscopy and its interaction with the cytoskeleton in migrating cells. BM-MSC underwent scratch and they were treated or not with PDGF-AB. uPAR and phalloidin stainings were performed 6 hours after the scratch (this is the time at which cell migration, cytoskeleton remodeling, and lamellipodia extension begin). Immunofluorescence analysis revealed that in resting cells uPAR was uniformly distributed on the cell surface and dispersed as small aggregates. In migrating cells, PDGF-AB treatment strongly modified cell morphology and uPAR distribution, with a clustering of uPAR at the leading edge of the cells in focal adhesions (Additional file 7). We next examined the distribution of uPAR and β1 integrin on migrating cells induced by PDGF-AB. Because uPAR staining appeared to be partially co-localized with β1 integrin as soon as 1 hour after PDGF-AB treatment of BM-MSC plated on type I dermal collagen (Fig. 4a) and on fibronectin (Figure S7A in Additional file 8), we used coimmunoprecipitation experiments to determine whether uPAR and β1 integrin were physically associated. Results shown in Fig. 4b demonstrated that the antibodies against the β1-integrin subunit (but not the control IgG) precipitated not only the β1-integrin subunit but also uPAR in nontreated cells, suggesting a physical association between uPAR and the β1-integrin subunit. The quantity of coimmunoprecipitated β1 integrin and uPAR was increased 1 and 3 hours post PDGF-AB treatment. The same trend was obtained at 3 hours on fibronectin (Figure S7B in Additional file 8).

To evaluate whether the physical association of uPAR with β1 integrin was involved in PDGF-AB-induced cell migration, we performed a scratch assay on control or PDGF-AB-treated BM-MSC using β1P1 peptide that acts as a competitive inhibitor of β1 integrin. Results in Fig. 4c showed that β1P1, as well as anti-uPAR neutralizing antibodies, blocked PDGF-AB-induced wound closure by cells seeded on type I dermal collagen. The same results were obtained when the cells were seeded on fibronectin but not on vitronectin (Additional file 9). The scrambled control peptide had no effect independently of the matrix used. Taken together, these data demonstrate that PDGF-AB enhances uPAR interaction with β1 integrin to promote BM-MSC migration on dermal collagen and fibronectin but not on vitronectin.

### PDGF-AB increases the interaction of uPAR with the β1-integrin signaling pathway

**Fig. 5:**
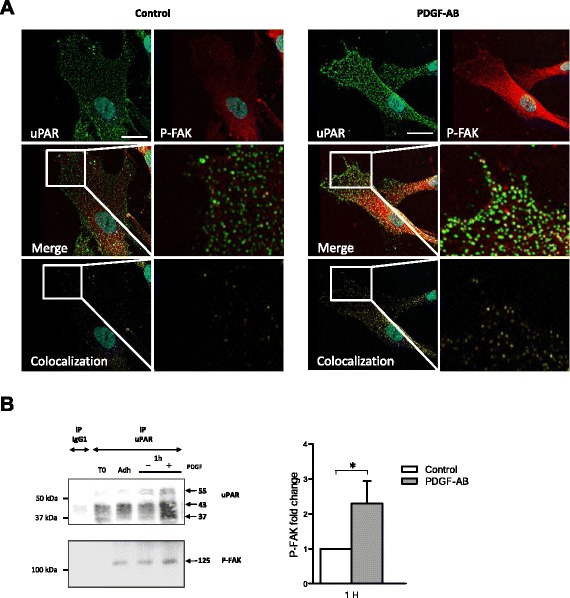
PDGF-AB enhances uPAR/P-FAK interactions in migrating BM-MSC. **a** uPAR (*green*) and P-FAK (*red*) repartition in migrating cells. BM-MSC were seeded in a Labtek chamber coated with type I dermal collagen. After the scratch was performed, cells were grown in serum-free control medium or treated with PDGF-AB for 3 hours. uPAR and P-FAK stainings were merged to show colocalization (*yellow*). Nuclei were stained with DAPI. Scale bars: 10 μm. **b** uPAR–P-FAK coimmunoprecipitation. Two hours after adherence on type I dermal collagen, cells were seeded in serum-free control medium (−) or treated with PDGF-AB (+) for 1 hour. uPAR was immunoprecipitated from cell lysates with anti-uPAR monoclonal antibodies or isotypic control IgG_1_. Immunoprecipitates were analyzed by SDS-PAGE and blotted for uPAR and P-FAK. (*Left*) Western blot representative of three experiments. T0, lysate of cells before seeding; Adh., lysate of cells allowed to adhere for 2 hours on type I dermal collagen. (*Right*) P-FAK blots quantification by densitometry analysis using ImageJ software. Data expressed as the P-FAK fold change after PDGF-AB treatment compared with serum-free control medium from three independent experiments (one donor per experiment). *PDGF-AB* platelet-derived growth factor AB, *P-FAK* autophosphorylated focal adhesion tyrosine kinase, *uPAR* urokinase-type plasminogen activator receptor

The focal adhesion tyrosine kinase (FAK), when autophosphorylated at Tyr^397^ (P-FAK), is known to be involved in the β1-integrin signaling pathway induced by uPAR. We thus examined uPAR and P-FAK expression in migrating cells on type I dermal collagen in response to PDGF-AB treatment. As for uPAR, P-FAK immunofluorescent staining was poorly detected in resting control cells, while it was clearly enhanced in PDGF-AB-induced migrating cells (Fig. 5a). To assess whether uPAR and P-FAK were physically associated in focal complexes, coimmunoprecipitation experiments were performed on cell lysates from BM-MSC induced to migrate on type I dermal collagen. As shown in Fig. 5b, the anti-uPAR antibodies (but not the control IgG) immunoprecipitated uPAR isoforms as well as P-FAK in nontreated cells, suggesting a physical association between uPAR and P-FAK. This effect appeared to take place as soon as the cells adhered to the coated plastic and was enhanced after 1 hour of treatment with PDGF-AB.

### The PDGF-AB effect is not restricted to MSC derived from bone marrow

**Fig. 6:**
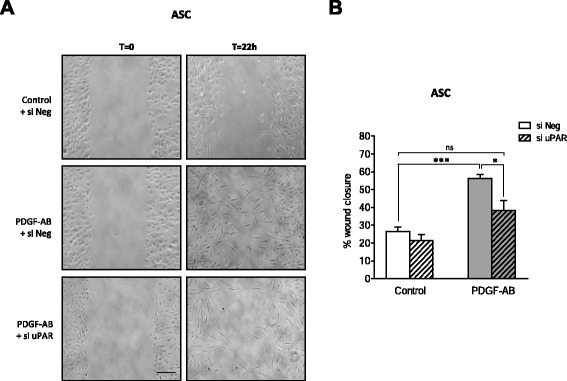
ASC migration is mediated by uPAR. **a** Scratch test assay was performed on ASC isolated from three different donors transfected with either si Neg or si uPAR and grown on type I dermal collagen in serum-free control medium or PDGF-AB-supplemented medium. Scale bars: 200 μm. **b** Quantification of ASC migration. Results expressed as percentages of wound closure at T = 22 h compared with T = 0 for each condition. Mean of three independent experiments ± SEM is represented (one donor per experiment), each performed in triplicate. *P <0.05. *ASC* adipose stem/stromal cells, *si Neg* nontargeting small interfering RNA, *si uPAR* uPAR-targeted small interfering RNA, *PDGF-AB* platelet-derived growth factor AB

We wondered whether uPAR was required for migration of MSC from other tissue origin such as ASC that can be isolated from adipose tissue. Migration assays were performed following PDGF-AB treatment and uPAR gene expression knockdown. ASC were transfected with control siRNA (si Neg) or uPAR targeted siRNA (si uPAR) and the knockdown efficiency was confirmed by quantitative RT-PCR and flow cytometry experiments that indicated, respectively, a 60 % decrease in uPAR mRNA expression (Figure S9A in Additional file 10) and a 90 % decrease in uPAR membrane protein expression (Figure S9B in Additional file 10). The scratch test assay was then conducted on type I dermal collagen in the presence or not of PDGF-AB. ASC migration showed a 30 % increase after 22 hours of treatment with PDGF-AB (Fig. 6a, b), while this increase observed under PDGF-AB treatment was inhibited when ASC were transfected with uPAR siRNA. On transwell dishes, PDGF-AB enhanced by sevenfold the number of migrated ASC and this effect was significantly reduced by uPAR siRNA (52 % reduction) (Figure S9C in Additional file 10). Similar results were thus obtained with ASC and BM-MSC, showing that PDGF-AB increased ASC migration on type I dermal collagen and that this effect was partially abrogated by knocking down uPAR gene expression. These results demonstrated that uPAR is required for PDGF-AB-induced migration of MSC from different tissue origin (bone marrow and adipose tissue).

## Discussion

In response to inflammatory cytokines and growth factors that are released upon tissue injury, endogenous and grafted exogenous MSC have the potential to migrate towards the damaged areas and improve tissue regeneration. Among chemotactic factors, PDGFs are known to increase MSC migration through PDGF receptors (PDGFRs) [12, 40–42] but their effects are also mediated by different proteins such as thrombospontin that has been reported to be an important mediator of PDGF-DD-stimulated migration of retinal pericytes [14] and neuropilin-1 that has been shown to associate with PDGFRs signaling in the presence of PDGF-AA or PDGF-BB, thereby regulating MSC migration in a ligand-specific manner [43]. In the present study, we examined whether the uPA/uPAR system was involved in PDGF-AB-induced human MSC migration. First, we showed clear effects of PDGF-AB treatment on uPA and uPAR protein upregulation and activation in migrating human BM-MSC. Then, loss-of-function experiments using specific blocking antibodies and siRNA oligonucleotides provided strong evidence that the uPA/uPAR system is required for PDGF-AB to control migration of human MSC. Coimmunoprecipitation experiments confirmed that uPAR activation following PDGF-AB stimulation triggered uPAR downstream signaling, namely β1 integrin and FAK, an integrin-associated signaling molecule that plays a selective role in the regulation of cell migration induced by uPAR/β1 integrin [44–46]. In vascular smooth muscle cells, interplays between PDGF and the uPA/uPAR axis have been reported previously by Kiyan et al. [23], who demonstrated that uPA-induced migration signaling was transmitted through PDGFRs. The authors highlighted an association between the uPAR and PDGFRβ receptors induced by uPA stimulation. Previous data from Stepanova et al. [47] on smooth muscle cells described that PDGF-BB-induced cell migration required uPA, without demonstrating involvement of uPAR intracellular signaling, while Reuning et al. [48] demonstrated an increase in uPAR mRNA expression upon PDGF-BB treatment. Our data add new insights to these findings and provide evidence that PDGF downstream signaling involves the uPA/uPAR intracellular signaling in migrating MSC. Our results were obtained on dermal collagen and fibronectin but not on vitronectin, which is in agreement with Madsen et al. [49], who reported that uPAR can bind directly its two extracellular domains D2D3 to the glycoprotein vitronectin and that cell migration on vitronectin is positively regulated by uPA but does not require physical interaction between uPAR and β1 integrins.

The uPA/uPAR system is an ancient, highly conserved system that is crucial for many cell functions such as hemostasis, cell motility, invasion, proliferation, and survival. In the context of MSC engraftment or mobilization following tissue injury, MSC migration would be under the control of a stimulated autocrine loop involving uPAR. Supported by our complementary technical approaches on MSC from two different tissue origins, we propose that when vascular injuries that are associated with tissue lesions occurred, PDGF that is released from platelets and monocytes [50] would stimulate an autocrine loop that induces uPA increased expression and uPAR activation by uPA in MSCs. Activated uPAR would associate with β1 integrin and activate FAK signaling, thus triggering human MSC migration to the site of injury. This regulatory system of migration would be common to human bone marrow and adipose tissue mesenchymal cells. Other extracellular molecules such as vitronectin and the cleaved high molecular mass kininogen can bind to uPAR [32]; thus, it would be interesting to investigate whether PDGF-AB binds directly to uPAR or not. Supporting this hypothesis in another signaling system, Ball et al. [51] reported that vascular endothelial growth factor A (VEGF-A) could directly signal through PDGFRs to regulate human MSC migration. Elsewhere, we could consider that PDGF-AB may induce a cross-talk between PDGFRs and uPAR, leading to increased cell migration; in the same way as Kiyan et al. [23] highlighted an association of uPAR with PDGFRβ in an uPA-dependent manner.

## Conclusions

Our findings demonstrate a functional link between PDGF and the nonproteolytic uPAR signaling cascade that regulates human MSC migration. These findings provide new insights into the molecular basis of the PDGF-induced signaling mechanisms in human MSC for tissue remodeling.

## Additional files

Additional file 1: Figure S1. Showing human BM-MSC and ASC characterization. (PDF 229 kb) Additional file 2:
**Supplementary methods.** (DOCX 25 kb) Additional file 3: Figure S2. Showing PDGF-AB enhances BM-MSC migration but not proliferation. (PDF 130 kb) Additional file 4: Figure S3. Showing regulation of uPAR and uPA expression in BM-MSC by inflammatory cytokines. (PDF 54 kb) Additional file 5: Figure S4. Showing uPA/uPAR mediate PDGF-AB-induced BM-MSC migration on fibronectin and vitronectin. (PDF 45 kb) Additional file 6: Figure S5. Showing uPAR knockdown in BM-MSC. (PDF 135 kb) Additional file 7: Figure S6. Showing PDGF-AB effects on cell morphology and uPAR distribution. (PDF 51 kb) Additional file 8: Figure S7. Showing PDGF enhanced uPAR/β1-integrin subunit interactions in migrating BM-MSC. (PDF 299 kb) Additional file 9: Figure S8. Showing β1-integrin and BM-MSC migration. (PDF 39 kb) Additional file 10: Figure S9. Showing uPAR knockdown in migrating ASC. (PDF 134 kb)

## Abbreviations

ASC: Adipose stem/stromal cells
BM-MSC: Bone marrow mesenchymal stem cells
BSA: Bovine serum albumin
DAPI: 4',6-Diamidino-2-phenylindole
ECM: Extracellular matrix
EDTA: Ethylenediamine tetraacetic acid
FAK: Focal adhesion tyrosine kinase
FCS: Fetal calf serum
FGF-2: Fibroblast growth factor-2
HRP: Horseradish peroxidase
IL: Interleukin
MEMα: Minimum essential medium alpha
MSC: Mesenchymal stem cells
PBS: Phosphate-buffered saline
PDGF: Platelet-derived growth factor
PDGFR: Platelet-derived growth factor receptor
P-FAK: Autophosphorylated focal adhesion tyrosine kinase
PMA: Phorbol myristate acetate
PMSF: Phenylmethylsulfonyl fluoride
SEM: Standard error of the mean
siRNA: Small interfering RNA
TNFα: Tumor necrosis factor alpha
uPA: Urokinase-type plasminogen activator
uPAR: Urokinase-type plasminogen activator receptor
VEGF-A: Vascular endothelial growth factor A
VSMC: Vascular smooth muscle cells
