# Supplementary material for: Urokinase-type plasminogen activator receptor interaction with β1 integrin is required for platelet-derived growth factor-AB-induced human mesenchymal stem/stromal cell migration
Source: Stem Cell Res Ther. 2015 Sep 29;6:188. doi: 10.1186/s13287-015-0163-5 (PMC4588680; doi:10.1186/s13287-015-0163-5)
Supplement: Additional file 1: Figure S1. — Showing human BM-MSC and ASC characterization. (PDF 229 kb) [file 13287_2015_163_MOESM1_ESM.pdf]

Chabot V. et al; Figure S1

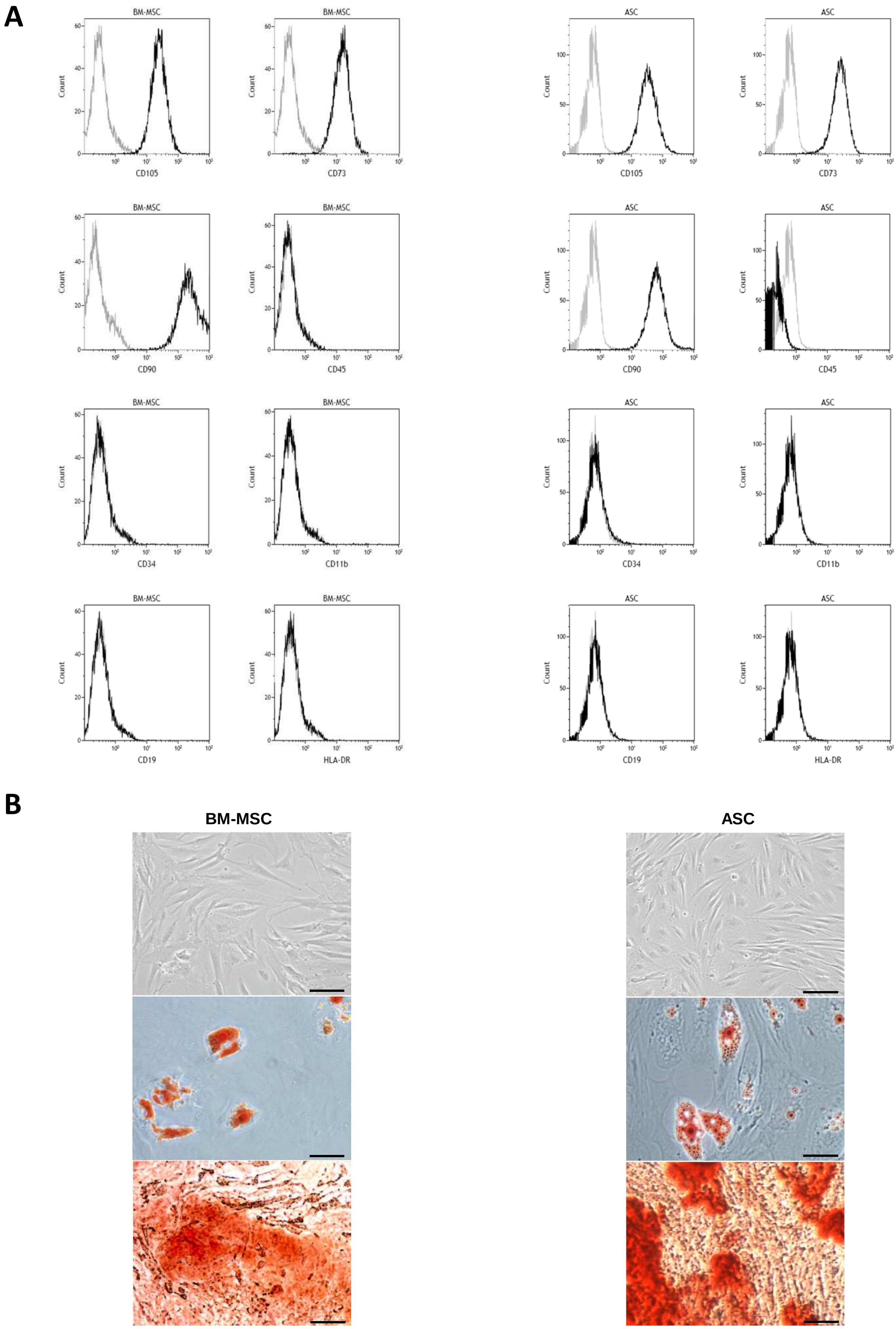

Figure S1: Human BM-MSC and ASC characterization . (A): Flow cytometry analysis. BM-MSC (left) and ASC (right) in culture display a mesenchymal phenotype: CD105+, CD73+, CD90+, CD45-, CD34-, CD11b-, CD19-, HLA-DR-. **(B):** BM-MSC (left) and ASC (right) adhere to plastic (upper panels) and differentiate into adipocytes demonstrated by numerous lipidic droplets stained with Oil red-O (middle panels) and osteoblasts demonstrated by calcic deposits stained with Alizarin red (down panels) when cultured under standard differentiating conditions. (scale bars 50 µm).
