# Supplementary material for: Urokinase-type plasminogen activator receptor interaction with β1 integrin is required for platelet-derived growth factor-AB-induced human mesenchymal stem/stromal cell migration
Source: Stem Cell Res Ther. 2015 Sep 29;6:188. doi: 10.1186/s13287-015-0163-5 (PMC4588680; doi:10.1186/s13287-015-0163-5)
Supplement: Additional file 2: — Supplementary methods. (DOCX 25 kb) [file 13287_2015_163_MOESM2_ESM.docx]

**Supplementary methods**

**Cell differentiation**

The day of plating was considered as day 0. Differentiation was assessed after 24 h (noted as day 1) and after 10 days or 21 days depending on differentiation lineage.

*Adipogenic differentiation*

On day 0, cells from dissociated spheres were plated at 65,800 cell/cm2 in 12-well tissue-culture plates (Falcon, Dominique Dutscher, Brumath, France) and cultured for 3 days in adipogenic differentiation medium which consisted in expansion medium supplemented with dexamethasone (1 μM), IBMX (450 μM), and indomethacin (60 μM) (Sigma Aldrich). Subsequently, IBMX was removed from the medium and differentiation was extended during 19 days. The medium was changed every 3 days. The extent of differentiation was noted by observation of multilocular refringent droplets in the induced cells and by staining of neutral lipids with Oil red-O.

*Osteogenic differentiation*

On day 0, cells from dissociated spheres were plated at 15,200 cell/cm2 in 12-well tissue-culture plates (Falcon) and cultured for 21 days in osteogenic differentiation medium which consisted in ASC expansion medium supplemented with dexamethasone (0.1 μM), ascorbic acid (250 μM), and NaH2PO4 (3 mM). The medium was changed every 4 days. Mineralization was revealed by staining calcium-rich deposits with Alizarin red.

**Transwell migration test**

Migration assays were also performed in transwell dishes (Corning Costar) 6.5 mm in diameter with 8 µm pore filters. The upper side of the transwell filter was coated for 1 hour at 37°C with type I dermal collagen from human skin (10 µg/ml, Merck Millipore-Calbiochem). P1 BM-MSC or ASC, each isolated from 3 donors, were added (1x 10^5^ cells) to the upper chamber and 600 µl of serum-free control medium with PDGF-AB (50 ng/ml) or not was added to the bottom chamber. After overnight incubation of the transwells at 37°C, 5% CO2, cells remaining on the upper face of the filters were removed with a cotton wool swab. Migration medium was replaced by 300 µl of Trypsin-EDTA 0.25% in the bottom chamber and transwells were incubated at 37°C for 5 min. Trypsin was neutralized by the addition of serum. The total number of cells that had migrated was counted using Malassez Cell and viability was assessed using Trypan blue. Data were expressed as percentages of cells related to that of the negative control.

**Proliferation Assay**

Cell proliferation was analyzed using a bromodeoxyuridine (BrdU) incorporation assay. BM-MSC were plated at 8.5 x 10^3^ cells/well in 96-well tissue culture plates (Corning) coated with human type I dermal collagen, cellular fibronectin or vitronectin. After 2 hours of adherence, they were grown in serum-free control medium supplemented with Bromodeoxyuridine (100 µM) +/- PDGF-AB for 22 hours at 37°C. BrdU uptake was measured using colorimetric Cell Proliferation ELISA (Roche Diagnostics, Meylan, France). BM-MSC without BrDU were used as the negative control. Duplicates were used for each condition and the experiments were repeated three times.

**Quantitative (Real-time) PCR**

Primer sequences:

- uPAR forward primer 5’-ATGGTTTCCACAACAACGAC-3’,
- uPAR reverse primer 5’-GGCAGATTTTCAAGCTCCAG-3’,
- uPA formard primer 5’-TTGTCCAAGAGTGCATGGT-3’,
- uPA reverse primer 5’-GTCTTTTGGCCACACTGAAA-3’
- GAPDH forward primer 5’-CTGGCGCTGAGTACGTCG-3’
- GAPDH reverse primer 5’-TTGACAAAGTGGTCGTTGA-3’

**Flow cytometry**

Dissociated spheres were incubated with PBS (phosphate-buffered saline) supplemented with 0.5% BSA and FcR Block reagent (StemCell Technologies, Vancouver). Stainings were performed by incubating 1 x 10^5^ BM-MSC or ASC for 30min at 4 °C with the following conjugated primary antibodies or appropriate IgG isotype controls: CD105-PE, CD73-PE, CD90-PE, CD45-PE, CD34-FITC, CD11b-FITC, CD19-FITC, HLA-DR-FITC (BD Biosciences, Le Pont de Claix, France). For detection of uPAR cell surface expression, cells were incubated on ice for 30-45 min with 0.5 µg of purified mouse anti-human CD87 antibody (clone VIM5) or purified mouse IgG1 control (both from BD Biosciences, Le Pont de Claix, France). The mAb VIM5 is directed against the uPAR binding site of uPA. We also used the Human uPAR antibody (clone 62022) and the mouse IgG1 control (both from R&D Systems, Lille, France). The mAb 62022 is directed against the D1 domain of uPAR. Same results were obtained with the VIM5 and 62022 clones. After washing with ice-cold PBS, cells were incubated with PE or A647-conjugated goat anti-mouse IgG antibody (BD Biosciences) on ice for 30 min. Labeled cells were analyzed on a FACS Calibur cytometer (BD Biosciences). Acquisition of at least 10,000 cells was performed and data were analyzed with the CELLQuest^®^ software (BD Biosciences). Results are expressed as the mean fluorescence intensity (MFI).

**Western blot analysis of BM-MSC cellular membranes**

uPAR and uPA expression was measured in BM MSC treated or not with PDGF-AB for 24 h. Membranes were isolated as described. A total of 5 µg of proteins was separated by 12% SDS-PAGE (sodium dodecyl sulfate polyacrylamide gel electrophoresis) and transferred to PVDF membranes for 1 h at 100 V. PVDF membranes were blocked overnight with 5% dried milk, incubated with either 1 µg/ml primary mouse monoclonal antibody (MAb) against uPAR (R & D systems), 1 µg/ml mouse monoclonal antibody against uPA (American Diagnostica, Neuville sur Oise, France) or β-actin antibody (1:5,000, Sigma-Aldrich) for 2 h at room temperature. PVDF membranes were washed and incubated with HRP-conjugated goat anti-mouse IgG (1:3,000, Bio-Rad, Marne la Coquette, France). Protein bands were revealed by chemiluminescence using the ECLplus Western blotting Reagent (GE Healthcare Europe GmbH, Aulnay sous Bois, France) and analyzed at 430 nm using a camera and Chemicapt^®^ software (Vilber Lourmat, Marne la Vallée, France). Bands were quantified by densitometry analysis using the ImageJ software (National Institute of Health, Bethesda).

**Zymography of BM-MSC cellular membranes**

BM-MSC cellular membrane proteins were isolated as described above and urokinase activity was determined by gelatin zymography using a standard methodology as we previously described [[23](#_ENREF_23)]. 5 µg of proteins were loaded into the wells of a 12% SDS polyacrylamide gel containing 1 mg/ml of gelatin (Prolabo, Fontenay-sous-Bois, France) and 22 µg/ml of human Glu-plasminogen (American Diagnostica). Purified high molecular weight human urokinase (American Diagnostica) was used as the standard. Electrophoresis was performed at 15 mA current for 2 h. Gels were incubated twice in 2.5% (v/v) Triton X-100 (Merck-Eurolab, Fontenay-sous-Bois, France) for 15 min to remove SDS. The Triton solution was replaced by developing buffer (50 mM Tris-HCl, pH 7.5, 5 mM CaCl_2_ and 0.02% sodium azide). After 24 hours of incubation at 37°C, gels were stained for 3 h in 30% ethanol, 10% acetic acid and 0.1% Coomassie brilliant blue R-350 (Amersham Biosciences, Chalfont St Giles, UK) and destained in 45% ethanol and 10% acetic acid. Areas of gelatin digestion were visualized as nonstained regions on the gel. Gels were analysed at 430 nm using a camera and ChemiCapt^®^ software (Vilber Lourmat). Bands were quantified by densitometry analysis using the ImageJ software (National Institute of Health, Bethesda).
