# Supplementary material for: Urokinase-type plasminogen activator receptor interaction with β1 integrin is required for platelet-derived growth factor-AB-induced human mesenchymal stem/stromal cell migration
Source: Stem Cell Res Ther. 2015 Sep 29;6:188. doi: 10.1186/s13287-015-0163-5 (PMC4588680; doi:10.1186/s13287-015-0163-5)
Supplement: Additional file 3: Figure S2. — Showing PDGF-AB enhances BM-MSC migration but not proliferation. (PDF 130 kb) [file 13287_2015_163_MOESM3_ESM.pdf]

Chabot V. et al; Figure S2

A

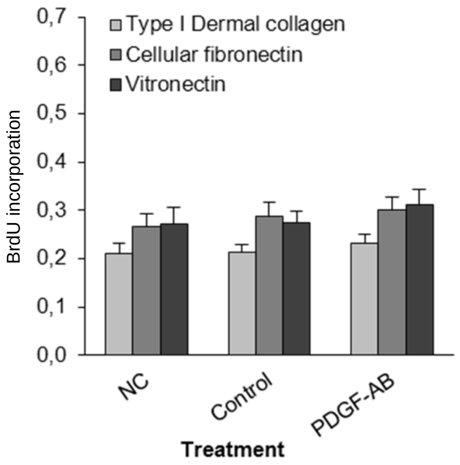

B

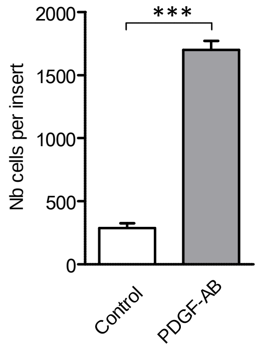

Figure S2 : PDGF-AB enhances BM-MSC Migration but not proliferation. **(A)**: BrdU incorporation in BM-MSC 22 hours after culture in serum-free control medium or PDGF-AB supplemented medium. BM-MSC without addition of BrDU were used as negative controls (NC). **(B)**: BM-MSC were seeded in collagen I coated-transwells. After 22h incubation, migrated cells on the lower face of the filters were counted. Data are expressed as mean ± SEM of total number of migrated cells per inserts, from three independent experiments (one donor per experiment), each performed in triplicate. ***P<0.001. Abbreviations: PDGF-AB, platelet derived growth factor AB, BrdU, bromodeoxyuridine.
