# Supplementary material for: Urokinase-type plasminogen activator receptor interaction with β1 integrin is required for platelet-derived growth factor-AB-induced human mesenchymal stem/stromal cell migration
Source: Stem Cell Res Ther. 2015 Sep 29;6:188. doi: 10.1186/s13287-015-0163-5 (PMC4588680; doi:10.1186/s13287-015-0163-5)
Supplement: Additional file 4: Figure S3. — Showing regulation of uPAR and uPA expression in BM-MSC by inflammatory cytokines. (PDF 54 kb) [file 13287_2015_163_MOESM4_ESM.pdf]

Chabot V. et al; Figure S3

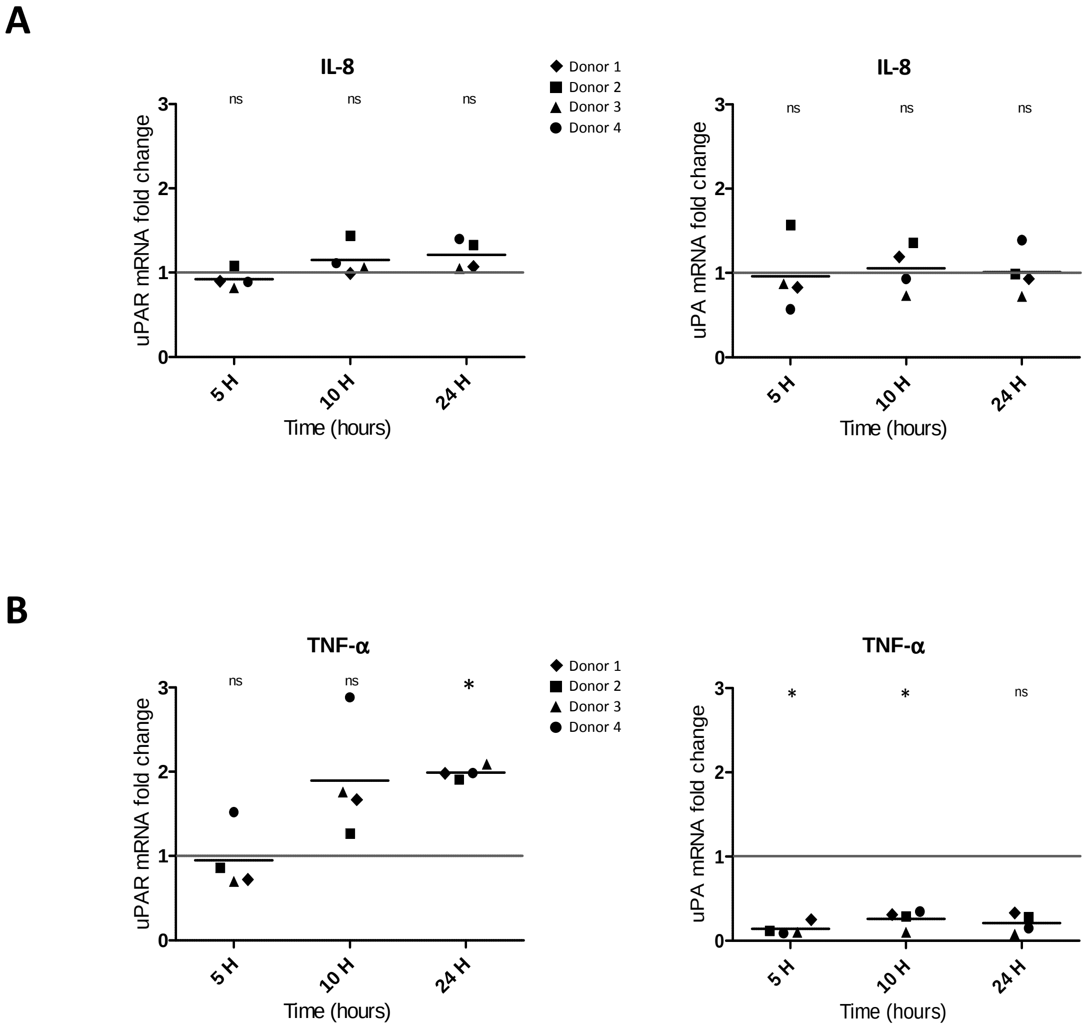

Figure S3: Regulation of uPAR and uPA expression in BM-MSC by inflammatory cytokines . BM-MSC isolated from four donors were grown in serum-free control medium or treated with IL-8 or TNF-α. **(A):** Quantitative PCR analysis of uPAR (left) and uPA (right) mRNA synthesis 5, 10 or 24 hours after treatment with IL-8. **(B):** Quantitative PCR analysis of uPAR (left) and uPA (right) mRNA synthesis 5, 10 or 24 hours after treatment with TNF-α. Data from four independent donors are presented as mRNA fold change relative to non-stimulated control cells for each donor (1= no stimulation). * $P<0,05$.
