# Supplementary material for: Urokinase-type plasminogen activator receptor interaction with β1 integrin is required for platelet-derived growth factor-AB-induced human mesenchymal stem/stromal cell migration
Source: Stem Cell Res Ther. 2015 Sep 29;6:188. doi: 10.1186/s13287-015-0163-5 (PMC4588680; doi:10.1186/s13287-015-0163-5)
Supplement: Additional file 5: Figure S4. — Showing uPA/uPAR mediate PDGF-AB-induced BM-MSC migration on fibronectin and vitronectin. (PDF 45 kb) [file 13287_2015_163_MOESM5_ESM.pdf]

Chabot V. et al; Figure S4

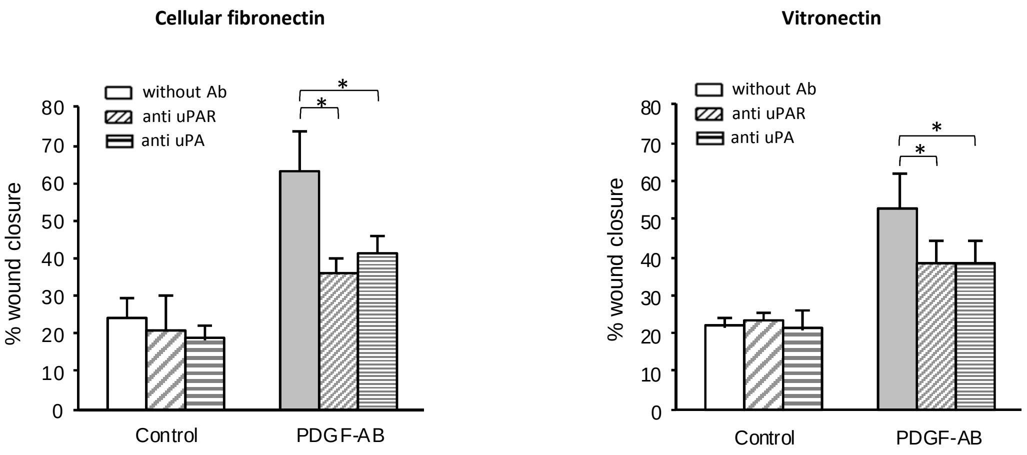

Figure S4. uPA/uPAR mediate PDGF-AB-induced BM-MSC migration on fibronectin and vitronectin. Scratch test assay was performed on BM-MSC isolated from three donors and seeded in plates coated with type cellular fibronectin (left) or vitronectin (right) and grown in serum-free control medium or PDGF-AB supplemented medium, in presence or not of anti-uPAR or anti-uPA neutralizing antibodies. Results are expressed as percentages of wound closure at T=22 h compared to T=0 for each condition. Mean of three independent experiments ± SEM are represented (one donor per experiment), each performed in triplicate. * P<0,05.
