# Supplementary material for: Urokinase-type plasminogen activator receptor interaction with β1 integrin is required for platelet-derived growth factor-AB-induced human mesenchymal stem/stromal cell migration
Source: Stem Cell Res Ther. 2015 Sep 29;6:188. doi: 10.1186/s13287-015-0163-5 (PMC4588680; doi:10.1186/s13287-015-0163-5)
Supplement: Additional file 6: Figure S5. — Showing uPAR knockdown in BM-MSC. (PDF 135 kb) [file 13287_2015_163_MOESM6_ESM.pdf]

Chabot V. et al; Figure S5

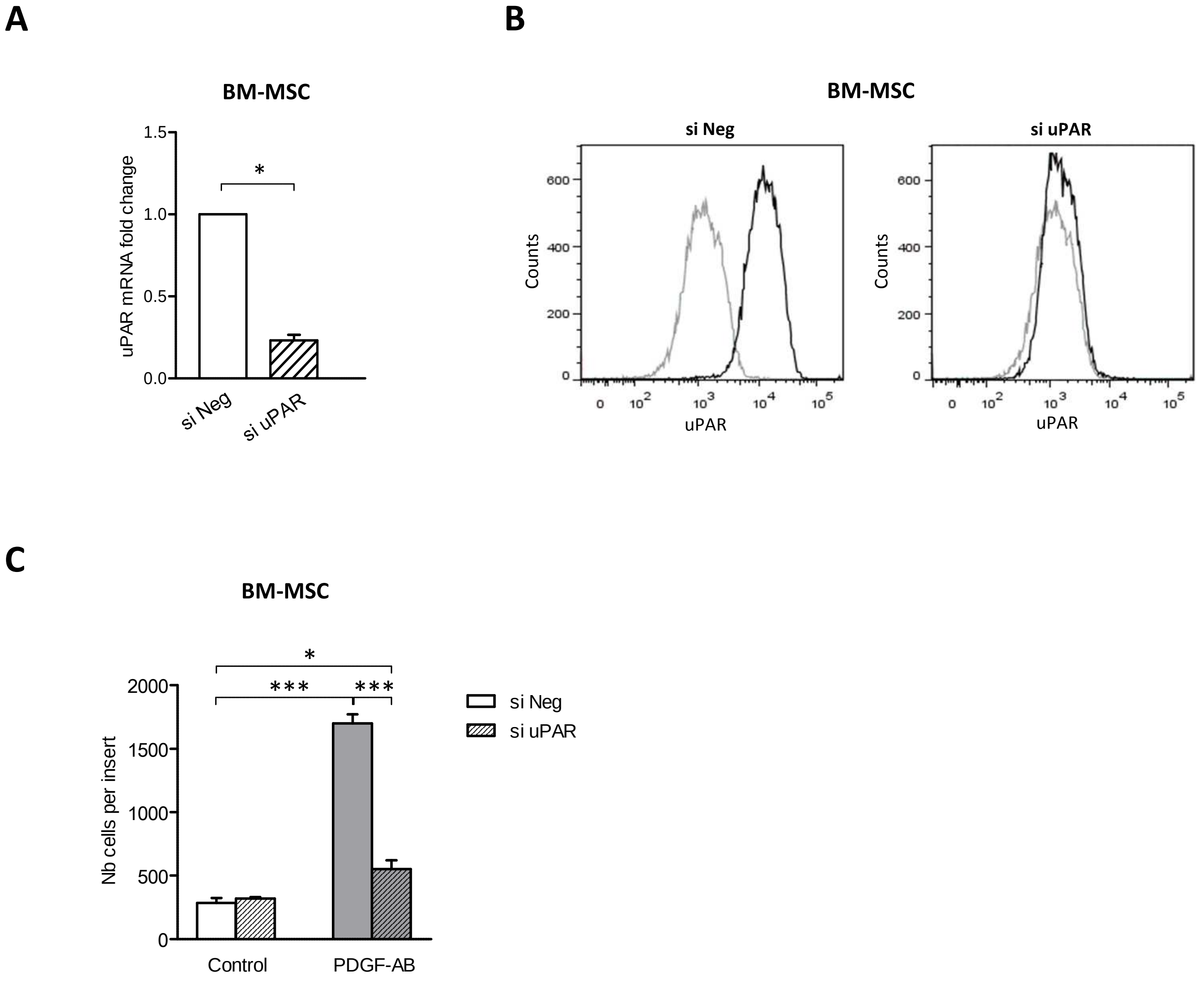

Figure S5 : uPAR knock down in migrating BM-MSC. (A): uPAR mRNA expression evaluation by qPCR on BM-MSC transfected with either si Neg or si uPAR. Results are expressed as uPAR fold change expression in cells transfected with si uPAR compared to si Neg. **(B):** Flow cytometric evaluation of uPAR protein expression in transfected BM-MSC. One experiment representative of the three experiments (one donor per experiment). **(C):** BM-MSC isolated from three donors were seeded on collagen I coated-transwells. After overnight incubation, migrated cells on the lower face of the filters were counted. Data are expressed as total number of migrated cells per inserts. Mean ± SEM of tree independent experiments are represented (one donor per experiment), each performed in triplicate. *P<0.05, ***P<0.001. Abbreviations: si Neg, non targeting small interfering RNA; si uPAR, uPAR targeted small interfering RNA.
