# Supplementary material for: Urokinase-type plasminogen activator receptor interaction with β1 integrin is required for platelet-derived growth factor-AB-induced human mesenchymal stem/stromal cell migration
Source: Stem Cell Res Ther. 2015 Sep 29;6:188. doi: 10.1186/s13287-015-0163-5 (PMC4588680; doi:10.1186/s13287-015-0163-5)
Supplement: Additional file 7: Figure S6. — Showing PDGF-AB effects on cell morphology and uPAR distribution. (PDF 51 kb) [file 13287_2015_163_MOESM7_ESM.pdf]

Chabot V. et al; Figure S6

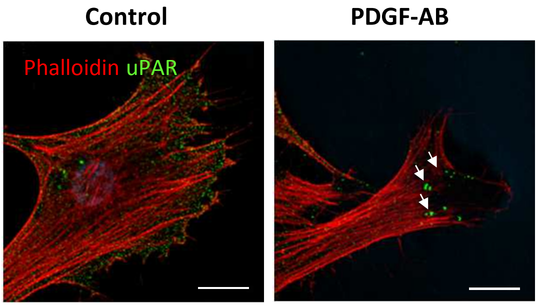

Figure S6 : PDGF-AB effects on cell morphology and uPAR distribution. uPAR (green) and phalloïdin (red) repartition in migrating cells. BM-MSC were seeded in Labtek chamber coated with type I dermal collagen. After the scratch was performed, cells were grown in serum-free control medium or treated with PDGF-AB for 6 hours. Arrows indicate uPAR repartition. Scale bars, 10 µm.
