# Supplementary material for: Urokinase-type plasminogen activator receptor interaction with β1 integrin is required for platelet-derived growth factor-AB-induced human mesenchymal stem/stromal cell migration
Source: Stem Cell Res Ther. 2015 Sep 29;6:188. doi: 10.1186/s13287-015-0163-5 (PMC4588680; doi:10.1186/s13287-015-0163-5)
Supplement: Additional file 8: Figure S7. — Showing PDGF enhanced uPAR/β1-integrin subunit interactions in migrating BM-MSC. (PDF 299 kb) [file 13287_2015_163_MOESM8_ESM.pdf]

Chabot V. et al; Figure S7

A

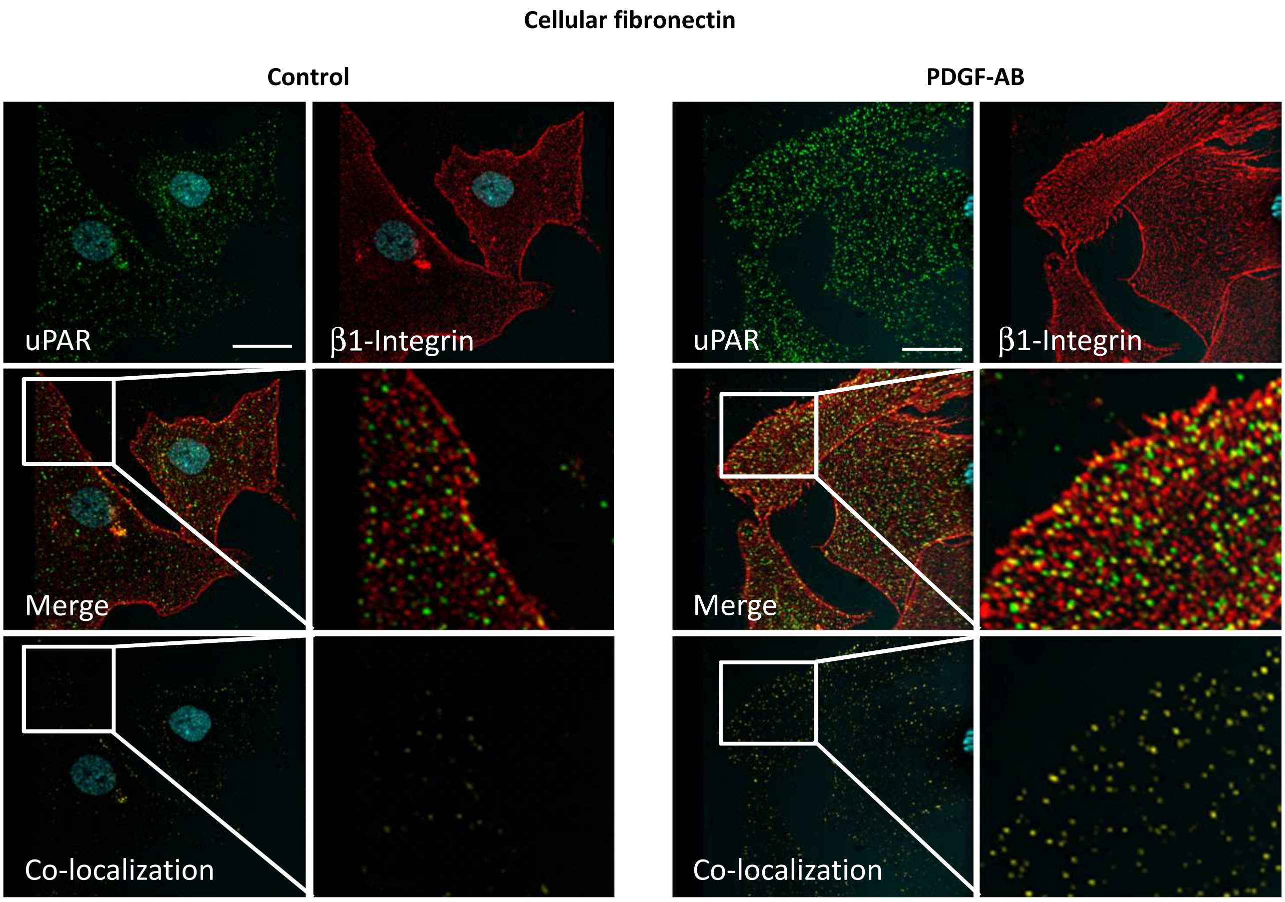

B

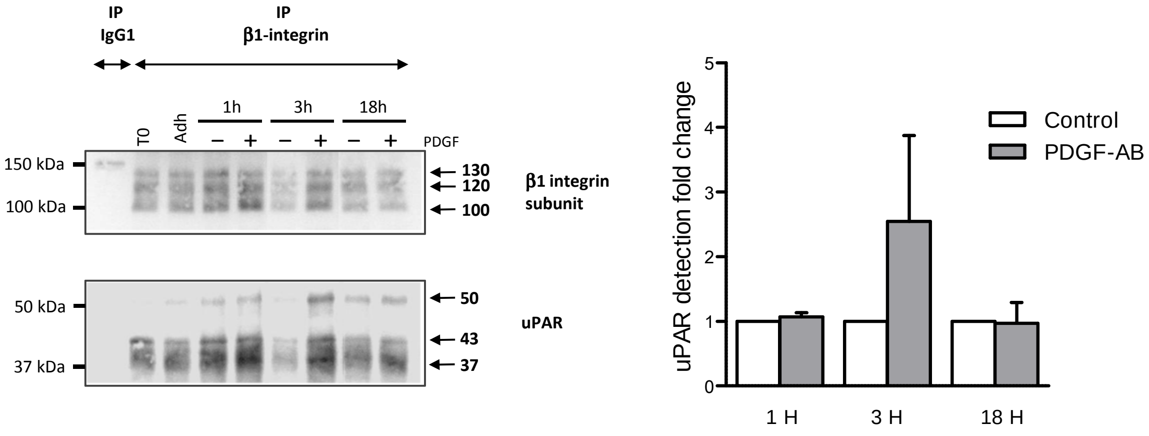

Figure S7: PDGF enhanced uPAR/β1 integrin subunit interactions in migrating BM-MSC. (A) uPAR (green) and integrin β1-subunit (red) staining on BM-MSC. BM-MSC were isolated from three donors and seeded in Labtek chamber coated with 5 µg/ml cellular fibronection. After the scratch was performed, cells were grown in serum-free control medium or treated with 50 ng/ml PDGF-AB for 1 hour. Cell staining was analyzed with a confocal microscope. uPAR and β1-integrin stainings were merged to show co-localization (yellow). Nuclei were stained with DAPI. Scale bars, 10 µm. **(B)** uPAR-β1 subunit co-immunoprecipitation. Two hours after adherence (Adh.) on cellular fibronectin, cells were grown in serum-free medium (-) or treated with 50 ng PDGF-AB (+) for 1 hour to 18 hours. Integrin β1-subunit in cell lysate was immunoprecipitated with anti-β1 monoclonal antibodies or isotypic control Igg. Immunoprecipitates were analyzed by SDS-PAGE and blotted for β1-subunit and uPAR. Data representative of three independent experiments are shown (one donor per experiment).
