# Supplementary material for: Urokinase-type plasminogen activator receptor interaction with β1 integrin is required for platelet-derived growth factor-AB-induced human mesenchymal stem/stromal cell migration
Source: Stem Cell Res Ther. 2015 Sep 29;6:188. doi: 10.1186/s13287-015-0163-5 (PMC4588680; doi:10.1186/s13287-015-0163-5)
Supplement: Additional file 9: Figure S8. — Showing β1-integrin and BM-MSC migration. (PDF 39 kb) [file 13287_2015_163_MOESM9_ESM.pdf]

Chabot V. et al; Figure S8

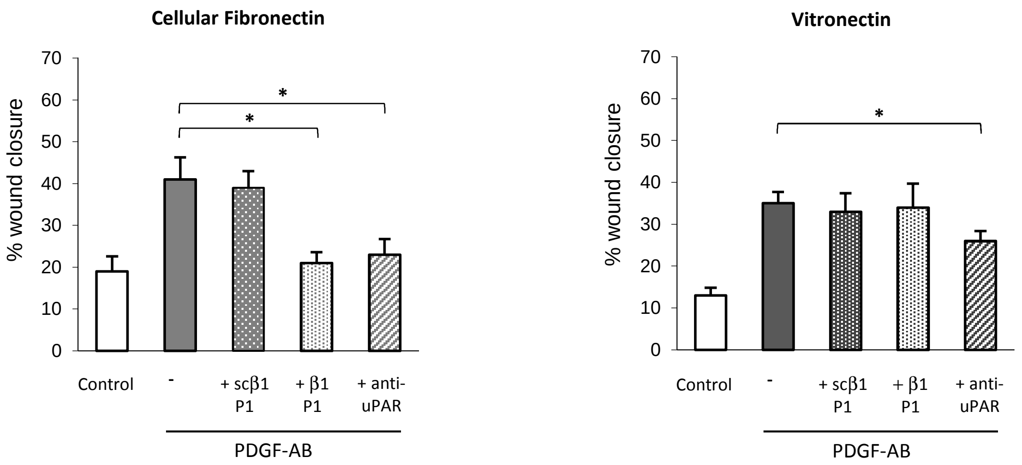

Figure S8 : β1 integrin and BM-MSC migration. Scratch test assay was performed on BM-MSC isolated from three donors and seeded in plates coated with type cellular fibronectin or vitronectin. Two hours after adherence, cells were cultured in serum-free control medium or treated with PDGF-AB, in presence or not (-) of integrin blocking peptide β1P1 along with the corresponding scrambled control scβ1P1 or anti-uPAR neutralizing antibody. Results are expressed as percentages of wound closure at T=22 h compared to T=0. Mean of three independent experiments ± SEM are represented (one donor per experiment), each performed in triplicate. * $P<0{,}05$.
