# Supplementary material for: Urokinase-type plasminogen activator receptor interaction with β1 integrin is required for platelet-derived growth factor-AB-induced human mesenchymal stem/stromal cell migration
Source: Stem Cell Res Ther. 2015 Sep 29;6:188. doi: 10.1186/s13287-015-0163-5 (PMC4588680; doi:10.1186/s13287-015-0163-5)
Supplement: Additional file 10: Figure S9. — Showing uPAR knockdown in migrating ASC. (PDF 134 kb) [file 13287_2015_163_MOESM10_ESM.pdf]

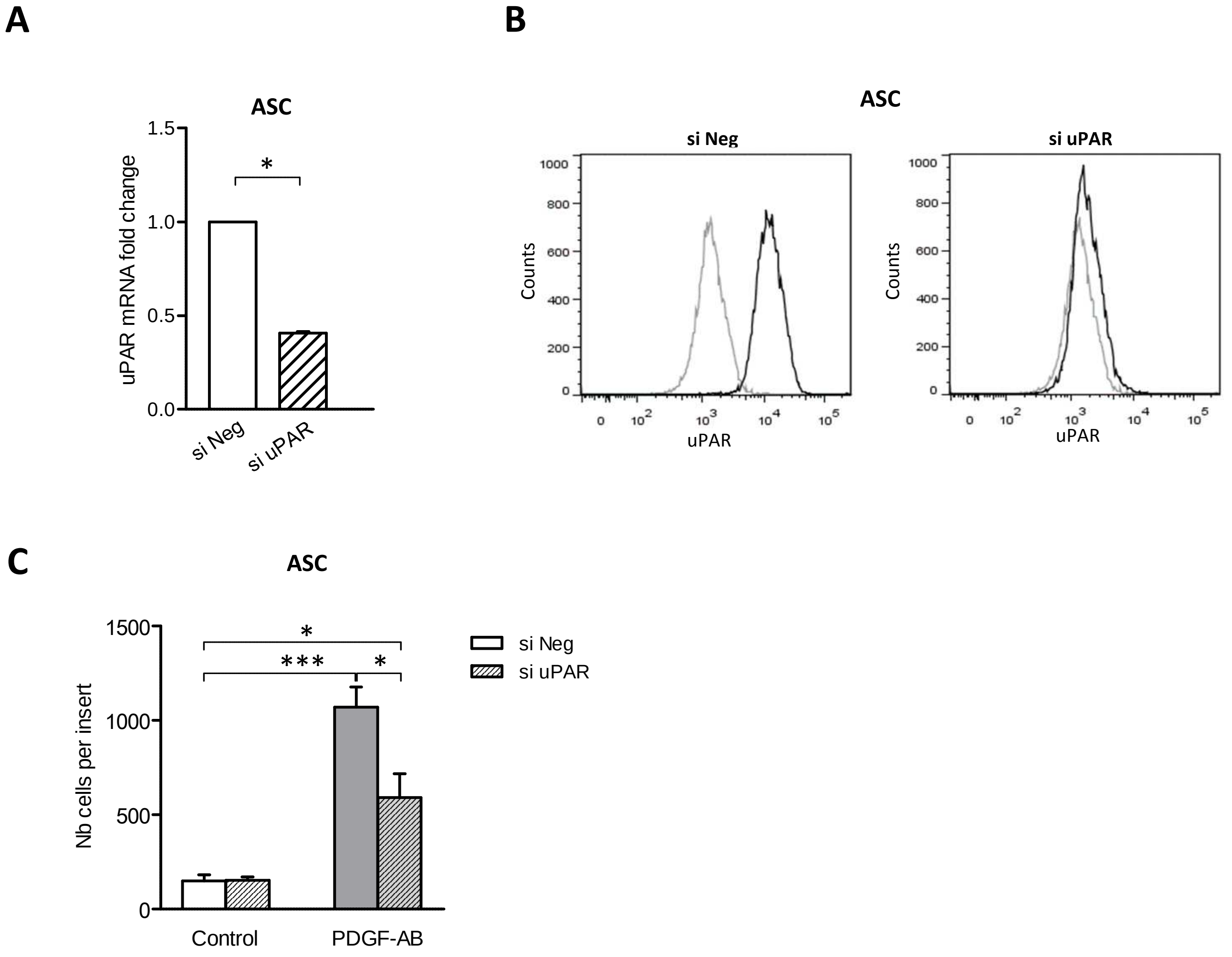

Figure S9 : uPAR knock down in migrating ASC. (A): uPAR mRNA expression evaluation by qPCR on ASC transfected with either si Neg or si uPAR. Results are expressed as uPAR fold change expression in cells transfected with si uPAR as compared to si Neg. **(B):** Flow cytometric evaluation of uPAR protein expression in transfected ASC. **(C):** ASC isolated from three donors were seeded on collagen I coated-transwells. After overnight incubation, migrated cells on the lower face of the filters were counted. Data are expressed as total number of migrated cells per inserts. Mean ± SEM of tree independent experiments are represented (one donor per experiment), each performed in triplicate. *P<0.05, ***P<0.001. Abbreviations: si Neg, non targeting small interfering RNA; si uPAR, uPAR targeted small interfering RNA.
